# Transformation and expressional studies of *GaZnF* gene to improve drought tolerance in *Gossypium hirsutum*

**DOI:** 10.1038/s41598-023-32383-0

**Published:** 2023-03-28

**Authors:** Fatima Batool, Sameera Hassan, Saira Azam, Zunaira Sher, Qurban Ali, Bushra Rashid

**Affiliations:** 1 National Centre of Excellence in Molecular Biology, University of the Punjab Lahore, 87 West Canal Bank Road Thokar Niaz Baig, Lahore, 53700 Pakistan; 2 Department of Plant Breeding and Genetics, Faculty of Agricultural Sciences, University of the Punjab Lahore, Lahore, Pakistan

**Keywords:** Biotechnology, Plant biotechnology, Molecular engineering in plants

## Abstract

Drought stress is the major limiting factor in plant growth and production. Cotton is a significant crop as textile fiber and oilseed, but its production is generally affected by drought stress, mainly in dry regions. This study aimed to investigate the expression of Zinc finger transcription factor’s gene (*GaZnF*) to enhance the drought tolerance in *Gossypium hirsutum*. Sequence features of the *GaZnF* protein were recognized through different bioinformatics tools like multiple sequence alignment analysis, phylogenetic tree for evolutionary relationships, Protein motifs, a transmembrane domain, secondary structure and physio-chemical properties indicating that *GaZnF* is a stable protein. CIM-482, a local *Gossypium hirsutum* variety was transformed with *GaZnF* through *Agrobacterium-*mediated transformation method with 2.57% transformation efficiency. The integration of *GaZnF* was confirmed through Southern blot showing 531 bp, and Western blot indicated a 95 kDa transgene-GUS fusion band in transgenic plants. The normalized real-time expression analysis revealed the highest relative fold spatial expression of cDNA of *GaZnF* within leaf tissues at vegetative and flowering stages under drought stress. Morphological, physiological and biochemical parameters of transgenic cotton plants at 05- and 10-day drought stress was higher than those of non-transgenic control plants. The values of fresh and dry biomass, chlorophyll content, photosynthesis, transpiration rate, and stomatal conductance reduced in *GaZnF* transgenic cotton plants at 05- and 10-day drought stress, but their values were less low in transgenic plants than those of non-transgenic control plants. These findings showed that *GaZnF* gene expression in transgenic plants could be a valuable source for the development of drought-tolerant homozygous lines through breeding.

## Introduction

Global warming is the key factor that elevated various abiotic stresses like drought, salt, temperature and heavy metals etc., all over the world that not only limit the plant’s growth but also result in hostile reactions such as damage to membrane system, osmotic imbalance, low respiratory and photosynthetic rates^1,2^.The world is facing severe food scarcity under prolonged effects of different abiotic stresses^3,4^. Plants have responded differently to drought stress at different morphological, physiological, biochemical and molecular levels by adapting evolved protective mechanisms^5^.

Cotton is approximately cultivated on 2079 thousand hectares in 2020–2021 in Pakistan with reduction of 17.4% in cultivated area of cotton as compared to 2019–2020. Pakistan ranked 4th in cotton production and accounts for 0.6% GDP to its economy, hence, considered as “white gold”^6^. Drought stress severely affects the physiological and biochemical processes in cotton, which leads to small boll formation, squares to shed and consequently, low yield and poor fibre quality (Economic Survey of Pakistan 2021–2022). It is believed that in the next two or three decades, the temperature on earth will increase to a rising average rate of 0.2 °C per decade^7^. Global warming, coupled with a decrease in available irrigation water, will severely affecting cotton yield and production in the near future^8^.

Transcription factors (TFs) are key elements that interact with cis-acting elements of promoters of defense genes to enhance the tolerance against abiotic stresses in various plants^9,10^. Zinc finger (ZnF) proteins are a large and diverse family of TFs, characterized by the ZnF motif. The protein motif consists of cysteine (C) or histidine (H) residues coordinated with several zinc ions. Different unique and important TFs have been discovered at the molecular level that will increase the plant adaptation to drought stress^11^ by regulating the expression of stress-related genes^12,13^. A stress tolerant Zinc finger transcription factor gene (*GaZnF*) was previously isolated from *G. arboreum*^10^. This study was aimed to develop transgenic *G. hirsutum* cotton plants through *Agrobacterium-*mediated genetic transformation by employing *GaZnF.* Moreover, the expression of *GaZnF* will be significant to develop a sustainable cotton variety to tolerate drought stress conditions.

## Materials and methods

### Structural and functional analysis of *GaZnF*

*GaZnF* gene sequence that was already isolated from *Gossypium arboreum* in our lab Zahur et al*.*^14^, was retrieved from NCBI database (Accession # GQ169757.1). It has been confirmed that the experimental sample of cotton plants, including the collection of plants, complied with relevant institutional, national, and international guidelines and legislation with appropriate permission from the authorities of the National Centre of Excellence in Molecular Biology, University of the Punjab Lahore, Pakistan.

A total of 22 transcription factors (HD-Zf) related to stress tolerance were searched from the Plant Transcription Factor Database (http://planttfdb.gao-lab.org/). A phylogenetic tree was constructed using CLC sequence viewer8 software (https://clc-sequence-viewer.software.informer.com/8.0/). The MEME motif software was used to analyze the amino acids (https://meme-suite.org/meme/tools/meme). Eight species showing relevant proteins were obtained from NCBI Protein Blast database (https://blast.ncbi.nlm.nih.gov/Blast.cgi?PAGE=Proteins) and compared with *GaZnF* protein to draw an evolutionary tree by using CLC sequence viewer8 software. The ExPASy bioinformatics resource portal (https://www.expasy.org/) was used to analyze the physical and chemical properties of *GaZnF*, including its molecular weight, isoelectric point, hydrophilicity, and stability. The trans-membrane region of *GaZnF* was analyzed by the TMHMM Serverv.2.0 online tool (http://www.cbs.dtu.dk/services/TMHMM/). The N-terminal signal peptide of the *GaZnF* protein was predicted by SignalP4.1 Server online tool (http://www.cbs.dtu.dk/services/SignalP-4.1/). The binding motif of *GaZnF* was determined using the Plant TFDB database (http://planttfdb.gao-lab.org/), while the secondary structure of *GaZnF* protein was analyzed using the SOPMA online tool (https://npsa-prabi.ibcp.fr/cgi-bin/npsa_automat.pl?page=/NPSA/npsa_sopma.html). After obtaining the base sequence of the upstream promoter region of *GaZnF*, the cis-acting elements of promoter region were retrieved by using the PLACE database (https://www.dna.affrc.go.jp/PLACE/?action=newplace).

### Isolation of *GaZnF* from *Gossypium arboreum*

#### Growing of plant material

Local cotton variety (FDH-171) *Gossypium arboreum* was grown in greenhouse as routine agronomic practices. Two-month-old, plants were drought-stressed, as the water was withheld for ten days till the wilting symptoms have appeared.

#### Total RNA extraction and cDNA synthesis

Total RNA was isolated from drought-stressed leaf tissues through a procedure as described by Jaakola et al.^15^. Then cDNA was synthesized using the Revert Aid TM H- first strand synthesis kit (Fermentas accession # K1621).

### PCR amplification of *GaZnF*

The *GaZnF* fragment (accession # GQ169757.1) was amplified through PCR using forward primers *GaZnF-ZF* 5´-CCATGGACCTCATGATGAGACGG-3´ along with *Nco*I restriction site and reverse primer *GaZnF-ZR* 5´-AGCTCACCTTCATCCTCGACTCTTT-3´ along with *Bgl*II restriction site respectively. The Reaction mixture contained: 2 μl cDNA, 2.5μl10X PCR buffer, 2.5 μl each primer (10 μM), 2.5 μl dNTPs (1 mM), 1.5 μl MgCl_2_ (25 mM), 0.2 μl of 5 Unit Taq DNA polymerase and 13.8 μl sterile water under following conditions: Hot start 95 °C for 5 min, followed by 35 cycles of 30 s at 94 °C, 30 s at 60 °C, 1 min at 72 °C and a final extension at 72 °C for 8 min.

### Cloning of *GaZnF* in TA & *pCAMBIA-1301* vector

The PCR product was TA cloned into PCR 2.1 vector Invitrogen (accession # K 4500-01), and the sequence was confirmed through Big Dye TM Terminator v3.0 sequencing kit (Applied Bio System) with reported accession (GQ169757.1). After restriction digestion of TA vector, a fragment of *GaZnF* gene was released that was cloned upstream of *GUS* gene under control of *CaMV*35S promoter at *Nco*I and *Bgl*II restriction sites in digested *pCAMBIA-*1301 with the same restriction enzymes. The resultant *pGaZnF* plasmid was confirmed by restriction digestion with *Nco*I, *Bgl*II and PCR amplification with gene-specific primers as mentioned above.

### Transformation of *pGaZnF* in *Agrobacterium*

The confirmed *pGaZnF* plasmid was transformed into *Agrobacterium LBA*4404 competent cells and confirmed through colony PCR. *Agrobacterium* cells transformed with *pGaZnF* were grown in Luria Bertani (LB) medium containing rifampicin and kanamycin (50 mg/ml). Then, the cells were harvested and suspended in 10 ml of Murashige and Skoog (MS) broth^16^.

### Transient expression of *pGaZnF* in *Nicotiana tabacum*

Transient expression of GUS was observed in the agro-infiltrated leaf tissue of the potted plantlets of *Nicotiana tabacum*. The *pGaZnF* construct was electroporated in *Agrobacterium* competent cells (*LBA-*4404) by using Bio-Rad electroporation device (# 165-2105). Then the construct was Agro-infiltrated in 6-weeks-old leaves of *Nicotiana tabacum* for transient expression assay. For this purpose, the construct was grown in YEP broth at 28 °C for 48 h (180 rpm) supplemented with Kanamycin (50 mg/ml). The next day culture was 1:50 diluted, grown till an OD_600_ of 0.8, centrifuged and re-suspended in 1 ml of re-suspension solution (MS broth with 10 mM MES and 200 mM Acetosyringone). The mixture was incubated at room temperature for 2 h and infiltrated into intact leaves using a 1 ml syringe without a needle. After 3 days, GUS activity was observed for blue colour to confirm the transgene expression after overnight incubation of leaf at 37 °C in X-Gluc (Sigma) staining solution in 100 mM Na_2_HPO_4_/ KH_2_PO_4_ pH 7.0; 20% v/v Methanol.

### Transformation of *pGaZnF* in the local variety of cotton

The cotton variety CIM-482 (*G. hirsutum*) was transformed with *pGaZnF* by the *Agrobacterium-*mediated transformation system as reported previously^17^. The apical meristems of mature embryos (7000) were cut after removing the testa to uncover the cotyledonary leaves. The cut embryos were treated with the suspension of *Agrobacterium* inoculum on a rotary shaker for 1 h. After co-cultivation, embryos were implanted on Petri plates containing MS medium at 25 ± 2 °C for 72 h under a photoperiod of 16 h (100–120µmm^−2^ s^−1^). After germination (72 h), seedlings were shifted to MS medium containing Cefataxime (250 mg/ml) in test tubes. They were sub cultured after every two weeks to the fresh MS medium containing Cefataxime and kanamycin (50 mg/ml). After eight weeks, plants were shifted to root induction selection-free MS medium supplemented with IAA 1 mg/L and kept on sub-culturing for eight weeks. Then well-developed rooted transgenic plants were shifted to the soil pots containing a soil mixture composed of autoclaved clay peat + moss + sand in 1 + 1 + 1 as described by^18^. For acclimatization, pot plants were left in the culture room for two weeks and then shifted to green house for further hardening and growth. Temperature was 30 ± 2 °C with humidity was approximately 45–50% and a photoperiod of 14 h. The plants in pots were watered with normal tap water every alternate day.

## Molecular analysis of transgenic plants

### Genomic DNA isolation

Genomic DNA was extracted from transgenic and controlled cotton plants' leaves using the Cetyl-trimethyl ammonium bromide (CTAB) method^19^. The quality of extracted DNA was evaluated and calculated by separation on 0.8% agarose gel stained with 0.5–1 μg/ml ethidium bromide, and the purity was confirmed using a Nanodrop ND-1000 spectrophotometer.

### PCR amplification of *GaZnF* in transgenic cotton plants

*GaZnF* was detected through PCR by using internal primers. Genomic DNA of non-transgenic plants was used as a negative control, and *pGaZnF* construct as a positive control. The PCR mixture was prepared as follows: 1.5 μl of DNA template (25 ng), 2 μl of 10 × PCR buffer, 2 μl of 1 mM dNTPs, 2.5U of Taq polymerase, 2 μl of 10 mM forward primer *GaZnF-ZF* 5´- GATTGATGAATCATGATGGAGATAGTT-3´, 2 μl of 10 mM reverse primer *GaZnF-ZR* 5´-GGCACCCAATCCAATGACTA-3´ and water to a final volume of 20 μl. The PCR was performed using the following cycling conditions: initial denaturation at 95* °C* for 4 min, followed by 35 cycles of 94 °C for 30 s, 60 °C for 1 min, and 72 °C for 45 s, with a final extension at 72 °C for 10 min.

### Southern blot analysis

The genomic DNA (15 µg) was digested with *EcoR*I to conduct a Southern blot to confirm the integration of *GaZnF* gene in transgenic plants. The method was followed as described by Southern^20^.

### Total protein extraction and Western blot analysis

Fresh leaves of transgenic and non-transgenic cotton plants were ground into fine powder in liquid nitrogen. Then, 50 mM Tris–HCl and 2 mM PMSF were added for incubation overnight at 4 °C. After centrifugation, the crude protein in the supernatant was quantified through Bradford assay. Western blot using anti- His- Primary Antibody (1:7000; Santa Cruz; cat#sc-804). For Western blot, SDS-PAGE was run according to the procedure described by Laemli^21^ and semi-dry Trans blot (Bio-Rad) was used. Signal for respective band size was detected with anti-Rabbit alkaline phosphatase-conjugated secondary antibody (1:5000; Santa Cruz; cat# sc-2034) through NBT/BCIP substrate.

### Quantitative real time-PCR (qRT-PCR) of transgenic cotton plants

For the spatial and developmental expression analysis, plants were given drought stress by withholding water for 10 days. Total RNA was isolated from leaf, and stem samples at the vegetative and flowering stages and cDNA was synthesized as mentioned above. Then quantitative real-time PCR was performed using gene-specific primers of *GaZnF-* forward 5’-GATTGATGAATCATGATGGAGATAGTT-3’and reverse 5’-GGCACCCAATCCAATGACTA-3’ primers. *GAPDH* was used as an internal control using forward 5’-TGGGGCTACTCTCTCAAAGGGTTG-3’ and reverse 5’-TGAGAAATTGCTGAAGCCGAAA-3’ primers. The reaction mixture contained: cDNA (2 µl), forward and reverse primer (10 pmol) (1 µl each), 2 × SYBR green master mix (7.5 µl), ddDEPC H_2_O (up to 15 µl). Thermo-cycler program for real-time PCR was denaturation at 95 °C for 30 s, annealing at 60 °C for 30 s, and extension at 72 °C for 30 s for 40 cycles. The final extension was done at 72 °C for 10 min. Data interpretation for CT values of triplicate samples was done with SDS 3.1 software (ABI).

### Application of drought stress and morphological, physiological and biochemical analysis of transgenic plants

Based on the performance of transgenic plants in molecular analysis, three transgenic plants (named as PF0027, PF0039 and PF0054) were selected for further analysis and confirmation of the expression of the transgene. The drought stress was applied to five-month-old transgenic and non-transgenic plants by water withdrawal till wilting of leaves. Readings of different parameters of all transgenic (PF0027, PF0039 and PF0054) and non-transgenic cotton plants were taken by selecting three replicates at 5- and 10-day drought stress and compared with non-stressed plants as 0-day stress.

### Morphologic analysis

Morphological characteristics for plant growth analysis of transgenic cotton plants were carried out by measuring plant height, Root to shoot ratio, root-to-total plant weight ratio (RWR), stem-to-total plant weight ratio (SWR), leaf-to-total plant weight ratio (LWR) and fresh and dry biomass as compared to non-transgenic control cotton plants at 0, 5 and 10 days of drought stress. The height of plants was measured from the soil surface to the apex using a measuring tape. The shoot length was measured from apex to the base of the hypocotyl. The root length was measured from tip of the root to the hypocotyl base at day 0, 5 and 10 day of drought stress. Three plants from each transgenic and non-transgenic line were selected. The root to shoot length ratio was calculated for each plant by taking values and means of measurement. For data collection, the parameters like root, stem and leaf to total plant weight ratio for transgenic and non-transgenic control plants under drought stress were taken. Plants were unsoiled and the fresh weight in gram (g) per plant was taken. Then plants were wrapped in brown papers and kept at 80 °C for 48 h. After that, dry weight was measured in g per plant. Percent reduction in biomass was measured at 0, 5 and 10 days after drought stress.

### Physiological and biochemical analysis

Physiological characteristics like Leaf relative water content (LRWC), chlorophyll content, photosynthesis, transpiration rate, and stomatal conductance of transgenic and non-transgenic cotton plants were calculated at 0, 5 and 10 day of drought stress by taking means of three values of each parameter. The leaf relative water content was calculated by taking a leaf sample 0.1 g from each treatment according to the method described by^22^. The chlorophyll a and chlorophyll b (photosynthetic pigments) were calculated according to^23^. Infra-Red Gas Analyzer (IRGA)was used to measure the net photosynthesis rate, transpiration rate, and stomatal conductance. Biochemical characteristics like Proline and the total soluble sugars were estimated by following the method described by^24,25^, respectively. After completion of all the parameters’ analysis, seeds were collected and stored for the study of the next generation.

### Statistical analysis

Graph Pad Prism7 was used for statistical analysis by subjecting the data for 2-way analysis of variance (ANOVA) to find out the significant difference in the mean.

## Results

### Structural and functional analysis of *GaZnF* gene

**Figure 1:**
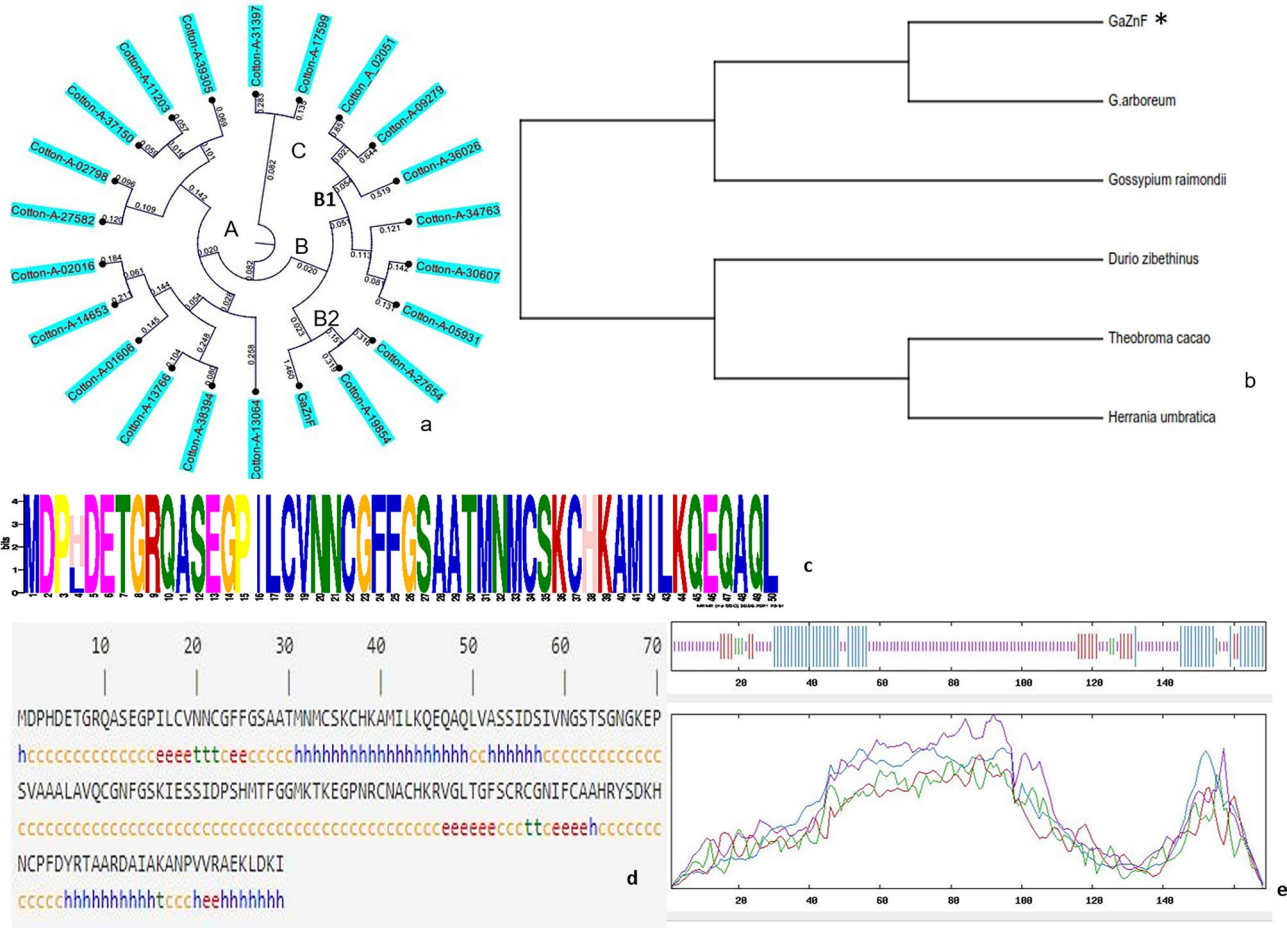
Structural and functional analysis of *GaZnF* gene. (**a**) Evolution analysis of HD-ZF family of *Gossypium arboreum*, (**b**) Clustering map of six specie’s protein (**c**) Analysis of *GaZnF* motif. The letters in the figure represent amino acids, (**d,e**) Secondary structure of *GaZnF* protein.

Clustering analysis of the ZF-HD family showed that 22 TFs were divided into 3 main subfamilies A, B and C. Family B is further divided into two subgroups (B1&B2). *GaZnF* belongs to subgroup B2 (Fig. 1a). The protein sequences of ZF-HD from 10 different species were obtained by NCBI alignment. It was found that *GaZnF* had a genetic relationship with *Gossypium raimondii* and Gossypium arboreum in the evolutionary process (Fig. 1b). Amino acid motifs retrieved from MEME suite software (Fig. 1c). The chemical formula of *GaZnF* is determined as C761H1219N233O240S17, the relative molecular weight is 18,017.49 Da and the theoretical isoelectric point was 8.66. The total counts of negatively charged residual bases (Asp + Glu) are 15, and the total counts of positively charged residual bases (Arg + Lys) are found to be 20. The fat coefficient of the peptide chain is 3605, and the instability coefficient is 2980. The instability index (II) is calculated to be 38.26. The above-mentioned characteristics classify the *GaZnF* protein as stable. Trans-membrane domain is found in the intramembrane protein encoded by *GaZnF* (Fig. 1Sa), and there is Meth signal peptide at the N-terminal of *GaZnF*. It is therefore speculated that the protein encoded by *GaZnF* is a non-secretory protein (Fig. 1Sb). Plant TFDB predicted the GaZnF protein binding motif (Fig. 1Sc). The secondary structure of the *GaZnF* protein contained 41.18% α helix (Hh), 4.62% extended chain (Ee), 1.68% β angle (Tt) and 52.52% irregular crimped (Cc) (Fig. 1d,e). Several motifs related to plant stress resistance are found in the 518 bp region upstream of *GaZnF,* including MYB transcription factors (CNGTTR motif) that regulate many stress responses, especially against drought stress in plants. The WRKY plant-specific transcription factor (motif TGAC) playing important roles in many different response pathways of diverse abiotic stresses (drought, saline, temperature, alkali and ultraviolet radiation) is a stress-related cis component (W-box).

### Cloning of *GaZnF* in TA & pCAMBIA-1301 vector

**Figure 2:**
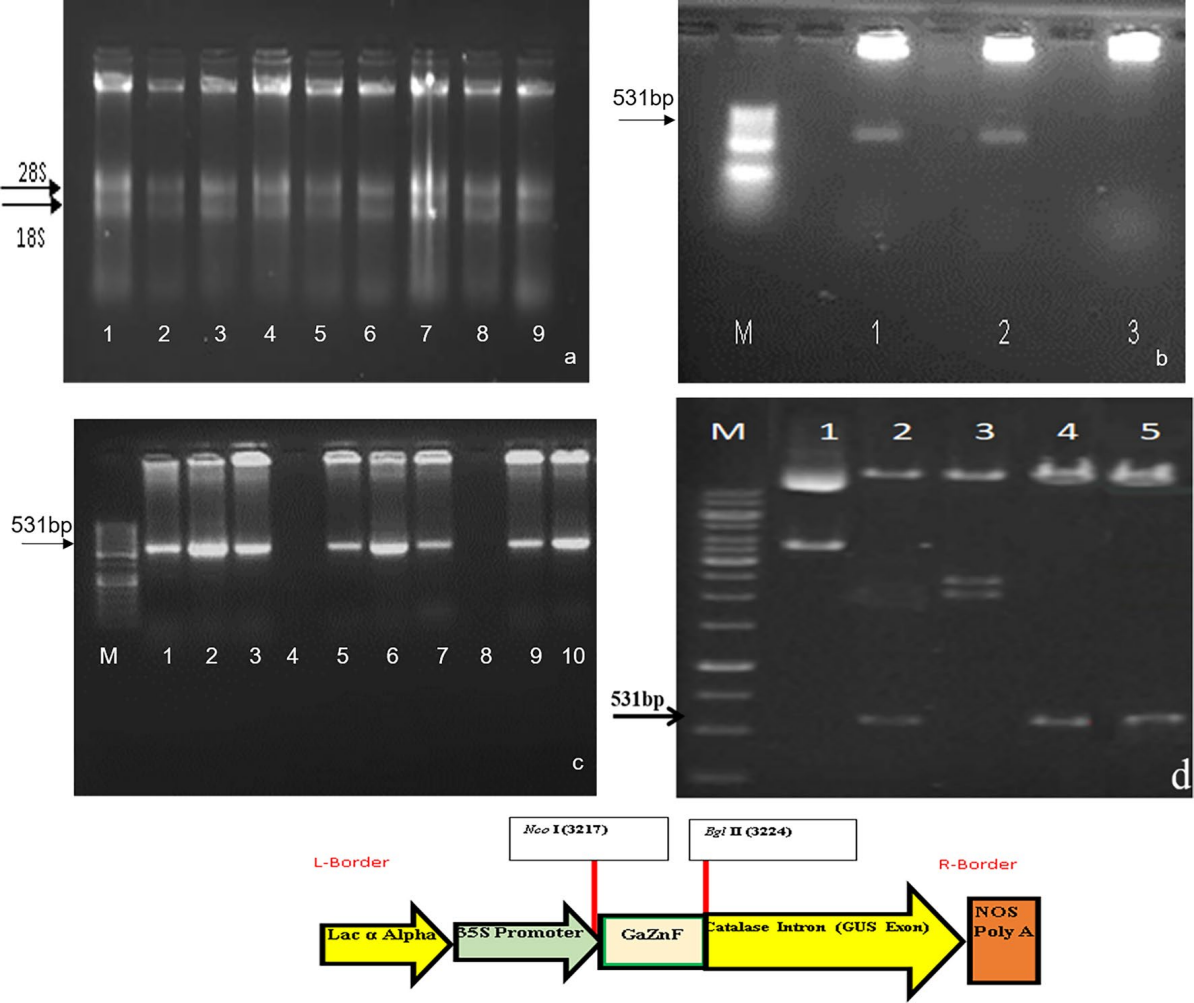
Cloning of GaZnF in TA & pCAMBIA-1301 vector. (**a**) Total RNA extracted from stressed *Gossypium arboreum*, (**b**) EcoRI Restriction Digestion of *GaZnF* cloned in TA vector, lane 1 & 2 fragment of *GaZnF* gene, lane 3 is negative control, Lane M is 50 bp marker of Thermo Fisher Scientific Cat# SM0613, (**c**) PCR Amplified fragment 531 bp of *GaZnF* cloned in pCAMBIA-1301 vector; lane 1 &2 no amplification of *GaZnF* gene in negative control , lane M is 50 bp marker of Thermo Fisher Scientific Cat# SM0613, (**d**) NcoI and Bgl II Restriction Digestion of *GaZnF* cloned in pCAMBIA-1301, lane 1 & 3 with negative control with empty vector pCAMBIA-1301, lane M is 1 Kb marker of Thermo Fisher Scientific Cat# SM1331, (**e**) Schematic representation of *GaZnF* clone in pCAMBIA-1301;* R* Right border,* L* Left Border,* NOS Poly A* nopaline synthase terminator,* 35S promoter* 35S cauliflower mosaic virus (CaMV).

cDNA synthesized by using total RNA from drought-stressed leaves was observed in Fig. 2a. A fragment of 531 bp released through restriction digestion by *EcoR*I confirmed the cloning of *GaZnF* in TA vector (Fig. 2b). The PCR amplification (Fig. 2c) with gene-specific primers of *GaZnF* and restriction digestion with *Nco*I and *Bgl*II (Fig. 2d) confirmed the cloning of gene in *pCAMBIA-*1301 vector. The graphical representation of full-length *GaZnF* gene is shown in (Fig. 2e).

### Transformation of *pGaZnF* in *Agrobacterium* and transient expression

**Figure 3:**
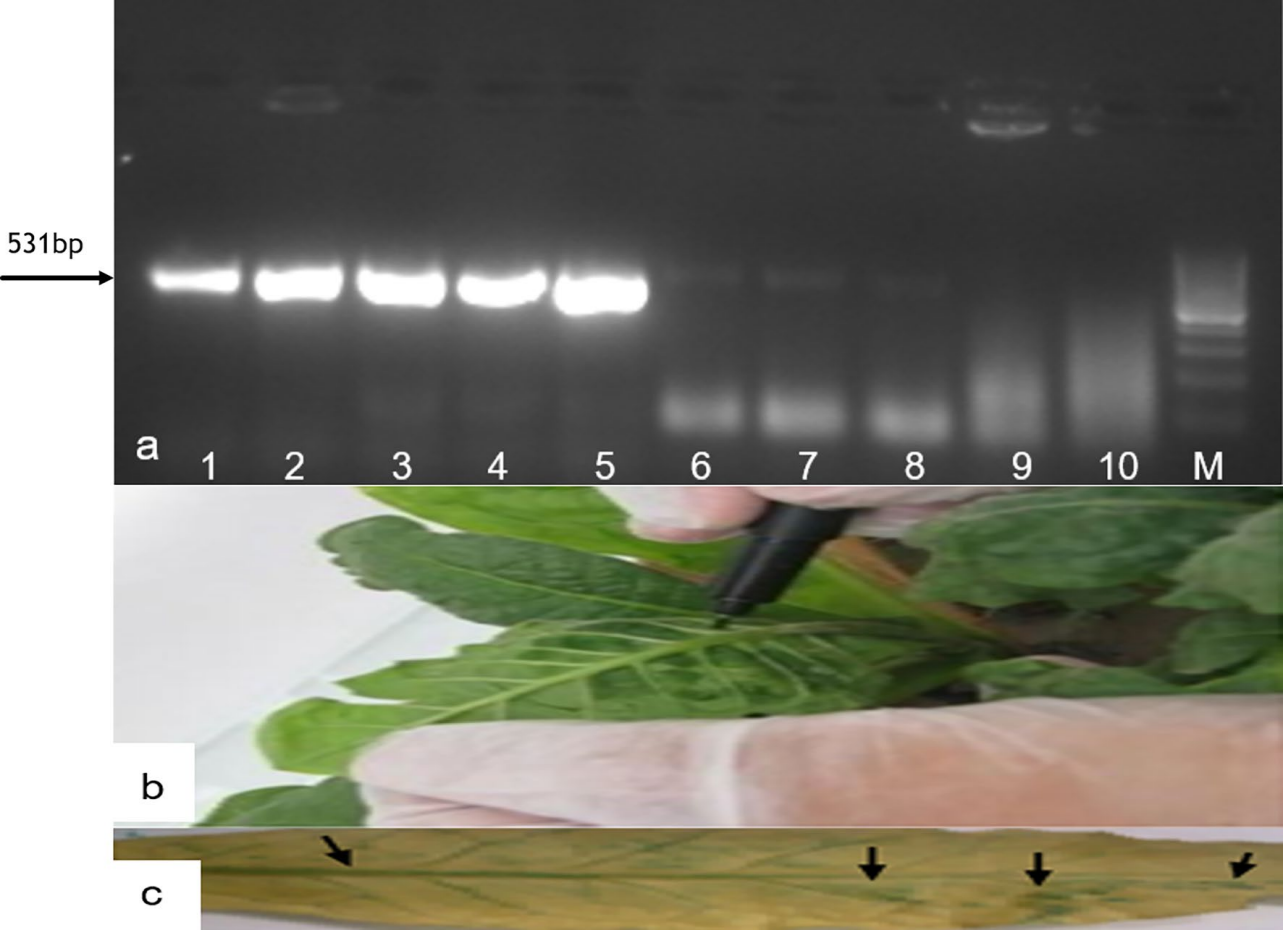
(**a**) Transformation of *pGaZnF* in *Agrobacterium*. (**a**) Colony PCR amplification of *GaZnF* gene, Lane M. 100 bp marker of Thermo Fisher Scientific Cat# SM1331, Lane 1. + ve control of *pCAMBIA-1301* with *GaZnF* gene, Lane 2–8. plasmid of colonies of Agrobacterium cells containing *GaZnF* gene, Lane 9–10. − ve control PCR amplification of empty *pCAMBIA-1301*. (**b,c**) Transient expression in Tobacco plants. (**b,c**) Agroinfiltration and histo-chemically GUS Assay in Tobacco species for transient expression of *GaZnF.*

Colony PCR with *GaZnF* gene specific primers confirms the transformation of *pCAMBIA35S-GaZnF* in Agrobacterium competent cells (Fig. 3a). The development of blue spots in the agro-infiltrated leaf confirmed the transient expression of GUS. The control leaf had no blue spots (Fig. 3b,c).

### Transformation of *pGaZnF* in local variety of cotton

**Figure 4:**
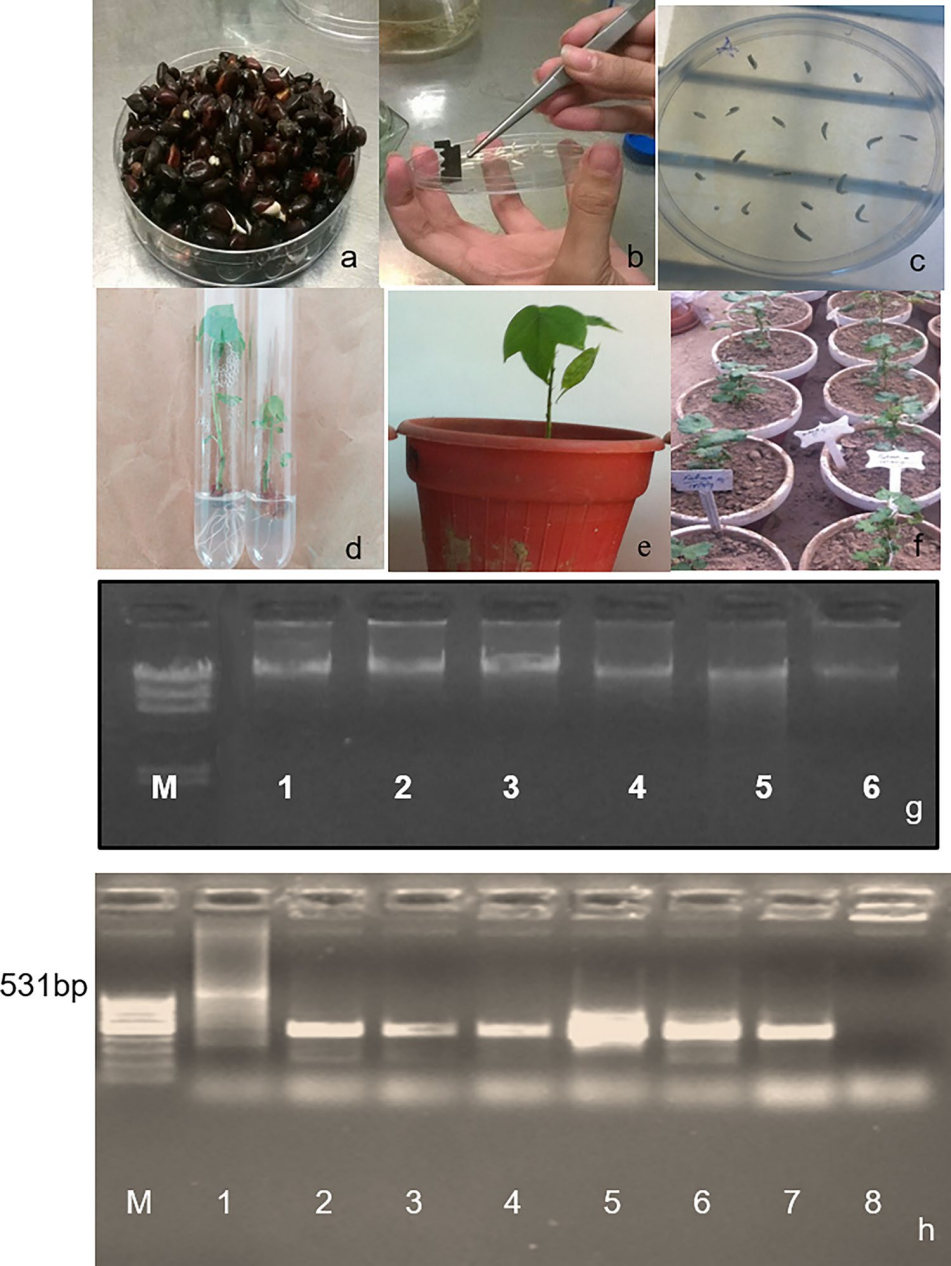
(**a-f**) *Agrobacterium* mediated Transformation of *GaZnF* in *Gossypium hirsutum* and Molecular analysis of transgenic plants. (**a**) Seed Germination, (**b**) A cut was given at apical meristem of isolated embryos from seeds and co-cultivation with Agrobacterium containing *GaZnF,* (**c**) Embryos on media containing plates, (**d**) Plants with developed shoots and roots, (**e**) Shifting of plants in soil containing pots, (**f**)shifting of pots containing plants in Green house for acclimatization. (**g,h**). Genomic DNA extraction and PCR amplification T0 generation. (**g**) Genomic DNA of transgenic and control plants, M. λ DNA/Hind III, Lane 1–6. Genomic DNA of *GaZnF* gene, (**h**) PCR amplification of genomic DNA of T0 generation’s transgenic and control cotton plants, Lane M was 100 bp Marker (Thermo scientific Cat. No.SM0241), Line 1,2,3,4,5 and 6 were Lane PF0022, PF0027, PF0031, PF0035, PF0039 and PF0054 respectively, Lane 7 was positive control and Lane 8 was negative control.

Local cotton variety CIM-482 (*G. hirsutum*) is selected for *GaZnF* gene transformation, and seeds of cotton variety (CEMB-482) are collected from CEMB Research Station Multan (30° 5′ 0″ N, 71° 40′ 0″ E) Punjab, Pakistan. During this study total of 7000 embryos were isolated (Fig. 4c), and co-cultivated with *Agrobacterium* containing *pGaZnF* by co-cultivation (Fig. 4a,b)., A total of 500 putative transgenic plants, are shifted to shoot-inducing medium. Out of these, 320 plants survived after 8 weeks, having well-developed shoots and roots in selection media. Non-transgenic plants are also shifted to shoot and root-inducing medium (Fig. 4d). Total of 180 putative transgenic plants with well-developed roots and shoots are shifted to soil pots. Non-transgenic plants are also shifted to soil pots (Fig. 4e,f). The overall efficiency of transformation is 2.57% (Table 1).

**Table 1:** Transformation efficiency of cotton embryos used in different experiments.

Total no. of embryos isolated	No. of plantlets survived on shoot inducing medium (6–8 weeks)	No. of plantlets survived on root inducing medium (3–4 weeks)	Total no. of plants shifted to soil (3 months old)	Transformation efficiency %
7000	500	320	180	2.57%

## Molecular analysis of transgenic plants

### PCR confirmation of genomic DNA

The amplification of the 531 bp product on 0.8% agarose gel in the genomic DNA confirmed the presence of the transgene in the transgenic plants. There is no amplification of transgene in non-transgenic control cotton plants (Fig. 4g,h).

### Southern & Western blot of transgenic plants

**Figure 5:**
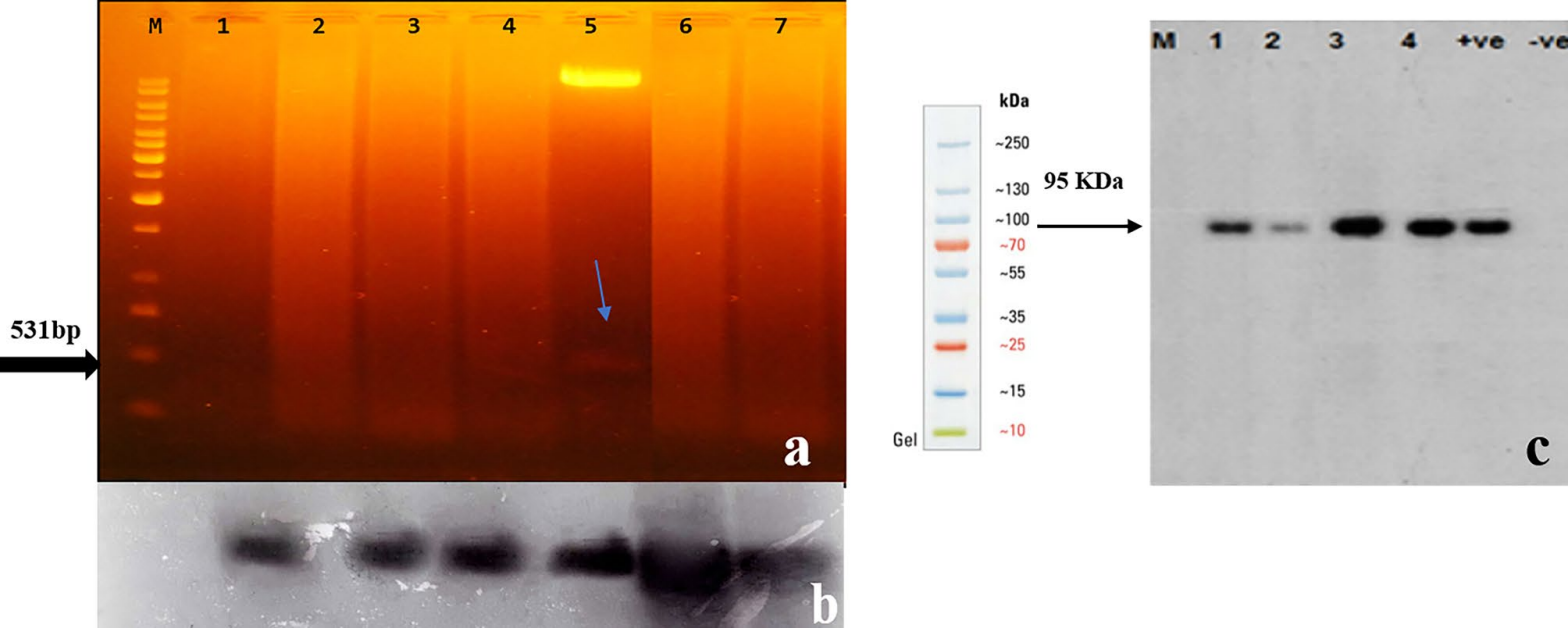
Confirmation of transgene (*GaZnF*) through southern and Western blot analysis. (**a,b**) Lane M. 1 kb DNA Ladder, Lane 1. Negative Control (DNA of untransformed plant), Lane 5 is positive control (plasmid DNA of *pCAMBIA-1301* with *GaZnF* gene), Lane 2, 3, 4 & 6, 7 (PF0027, PF0035, PF0039, PF0048 and PF0054 respectively). Selected Transgenic Cotton Plant Samples (*GaZnF*) were digested with *Nco I* and *Bgl II* probed with biotin-labeled *GaZnF* DNA to assess copy number of the transgene in the transformed plants. The presence of bands at size of positive control confirms the transgene’s integration in transgenic plants as compare to un-transformed plants that is used as negative control. Lane M contains 1 Kb ladder, lane 1 represent negative controls, lane 2, 4 and 6, 7 indicate the integration of *GaZnF* gene in selected transgenic cotton plant samples, (**c**) Lane –ve. total soluble protein of the non-transformed cotton plant, Lane + ve, purified *GaZnF* protein; Lanes 1–4, total soluble proteins of the transgenic cotton plants (PF0035, PF0039, PF0048 and PF0054).

Southern hybridization showed the integration via a 2.5 kb fusion band of *GaZnF*- GUS (Fig. 5a,b). While the transgene expression is confirmed by 95 kDa fusion band of *GaZnF*-GUS as detected by anti-His antibody in transgenic cotton plants (Fig. 5c).

### Quantitative real time-PCR (qRT-PCR) of transgenic cotton plants

**Figure 6:**
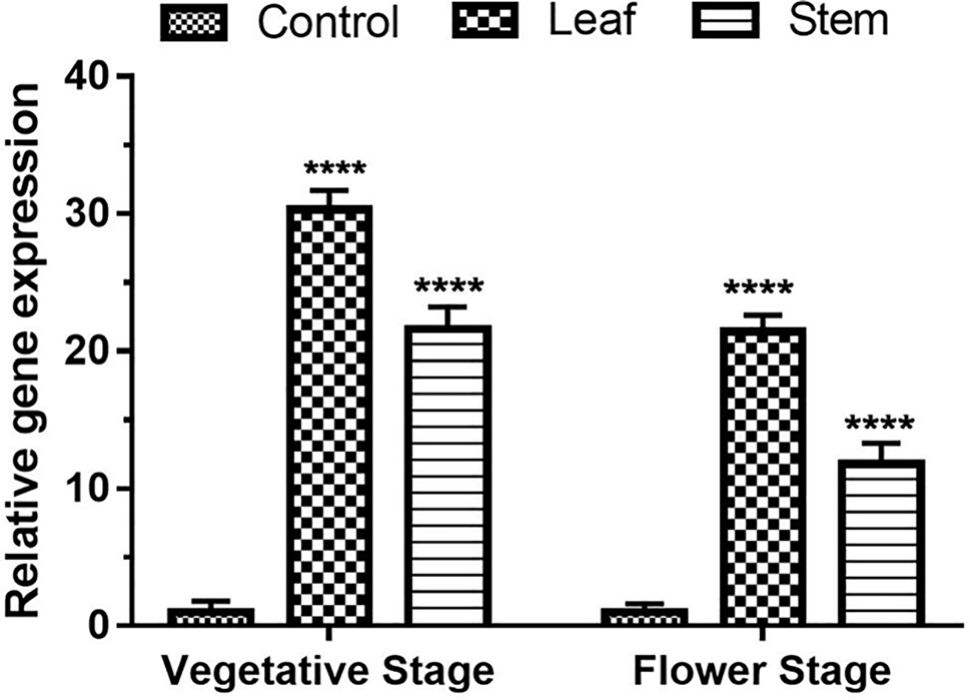
Quantitative real time PCR to determine the expression of transgenic cotton plants (*GaZnF*). mRNA samples of Line PF0054 exhibit the different expression levels at both vegetative and flower stages in leaf and stem tissues of transgenic and control plants, GAPDH was used as an internal control for normalization. The means ± SD of three biological replicates are presented. Error bars represent the standard deviation of the mean. Above the bars **** represent significant expression level of transcript in transgenic plants as compare to non- transgenic plants at the *P* < 0.05 level according to LSD multiple range test.

The spatial expression of *GaZnF* under drought stress is 30.3fold in the leaf and 21.6fold in stem of the transgenic plant compared to control plants at the vegetative stage. The *GaZnF* is also expressed strongly under drought stress, such as 21.4fold in leaf and 11.8fold in the stem compared to the control plants at the flowering stage. Overall, the maximum transcript abundance of *GaZnF* is recorded in the leaf tissue as compared to the stem (Fig. 6).

### Phenotypic analysis of transgenic cotton lines

**Figure 7:**
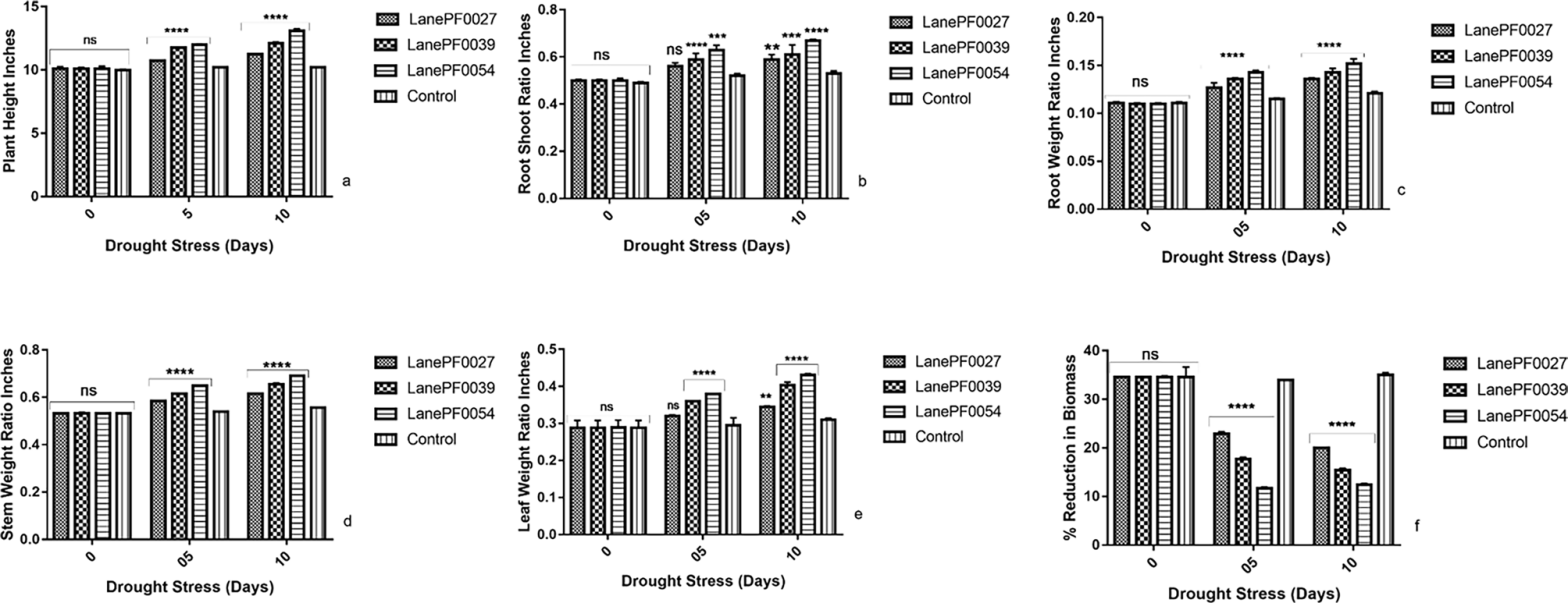
Effect of drought stress on growth indicators of transgenic cotton plants with *GaZnF* gene. (**a**) Comparison of plant height of transgenic and control plants, (**b**) Comparison of root shoot ratio of transgenic and control plants, (**c**) Comparison of root to total plant weight ratio of transgenic and control plants, (**d**) Comparison of stem to total plant weight ratio of transgenic and control plants, (**e**) Comparison of leaf to total plant weight ratio of transgenic and control plants, (**f**) Comparison of percentage of reduction in biomass of transgenic and control plants. The means ± SD of three biological replicates are presented. Error bars represent the standard deviation of the mean. Above the bars ****, ***, **represent significant and ns represent non-significant differences between transgenic and non-transgenic plants respectively at the *P* < 0.05 level according to LSD multiple range test.

Morphological parameters like plant height, root shoot ratio, root weight ratio, leaf weight ratio, stem weight ratio and percentage reduction in biomass were determined to observe the influence of the zinc finger gene (*GaZnF*) to enhance drought tolerance in transgenic cotton plants in the form of appearance as compared to non-transgenic control plants. The plant height is 10.72”,11.78” and 12.5” in PF0054, PF0027 and PF0039, respectively, at 5 day as compared to the non-transgenic control plant that is 10.19” while at 10 day is 11.25”, 12.1” and 13.1” in PF0054, PF0027 and PF0039 respectively as compare to non-transgenic control plant that is 10.21”. The acquired results showed that the plant height of Lane PF0054 increased by 27.18% as compared to non-transgenic control plants under drought stress (Fig. 7a). The root-to-shoot length ratio of transgenic cotton plants PF0054, PF0027 and PF0039 is recorded as 0.56”, 0.59” and 0.63” at 5 days as compare to non-transgenic control plant that is 0.52” while at 10 days ratio is increased to 0.59”, 0.61” and 0.67” in PF0054, PF0027 and PF0039 respectively as compare to non-transgenic control plant that is 0.12”. LanePF0054 showed prominent results as compare to other transgenic lines (Fig. 7b).The root to total plant weight ratio of transgenic plants PF0054, PF0027 and PF0039 is 0.127”, 0.136” and 0.143” respectively as compare to non-transgenic control plant that is 0.115” at 5 day of drought stress while transgenic lines PF0054, PF0027 and PF0039 showed an increase of 0.135”, 0.143” and 0.152” respectively as compare to non-transgenic control plant that is 0.12”at 10 day of drought stress (Fig. 7c). The stem to total plant weight ratio is 0.58”, 0.61” and 0.65” in PF0054, PF0027 and PF0039 respectively at 5 day of drought stress as compare to non-transgenic control plant that is 0.54” while at 10 day of drought stress treatment of PF0054, PF0027 and PF0039 is 0.615”, 0.655” and 0.69” respectively as compare to non-transgenic control plant that is 0.556” (Fig. 7d). The leaf to total plant weight ratio of PF0054, PF0027 and PF0039 is 0.32”, 0.36” and 0.38” respectively as compare to non-transgenic control plant that was 0.29” at 5 day of drought stress while at 10 day of drought stress the transgenic plants PF0054, PF0027 and PF0039 showed an increase of 0.345”, 0.403” and 0.431” respectively as compare to non-transgenic control plant that is 0.31” (Fig. 7e).An increasing trend was observed in plant height, root to shoot length ratio, root weight ratio, stem weight ratio and leaf weight ratio of transgenic plants as compare to non-transgenic control plants. Calculated values for % reduction in biomass, a decreasing trend observed in both the transgenic and non-transgenic plants with the increase in drought stress levels, but in the transgenic plants, the % reduction in biomass is less as compared to non-transgenic control plants. The % reduction in biomass of PF0054, PF0027 and PF0039 is 26.9%, 22.3% and 17.9%, respectively as compared to non-transgenic control plants that are 32.36% while transgenic plants PF0054, PF0027 and PF0039 showed a reduction in biomass as 22.00%, 18.15% and 12.4% respectively as compared to non-transgenic control plant that reduced to 30.58% at 10 day of drought stress (Fig. 7f). Two-way ANOVA indicated the significant difference between the plant height, root shoot ratio, root, stem and leaf to total plant weight ratio and % reduction in biomass of transgenic and non-transgenic genotypes (P < 0.05).

### Physiological and biochemical analyses of transgenic cotton plants

**Figure 8:**
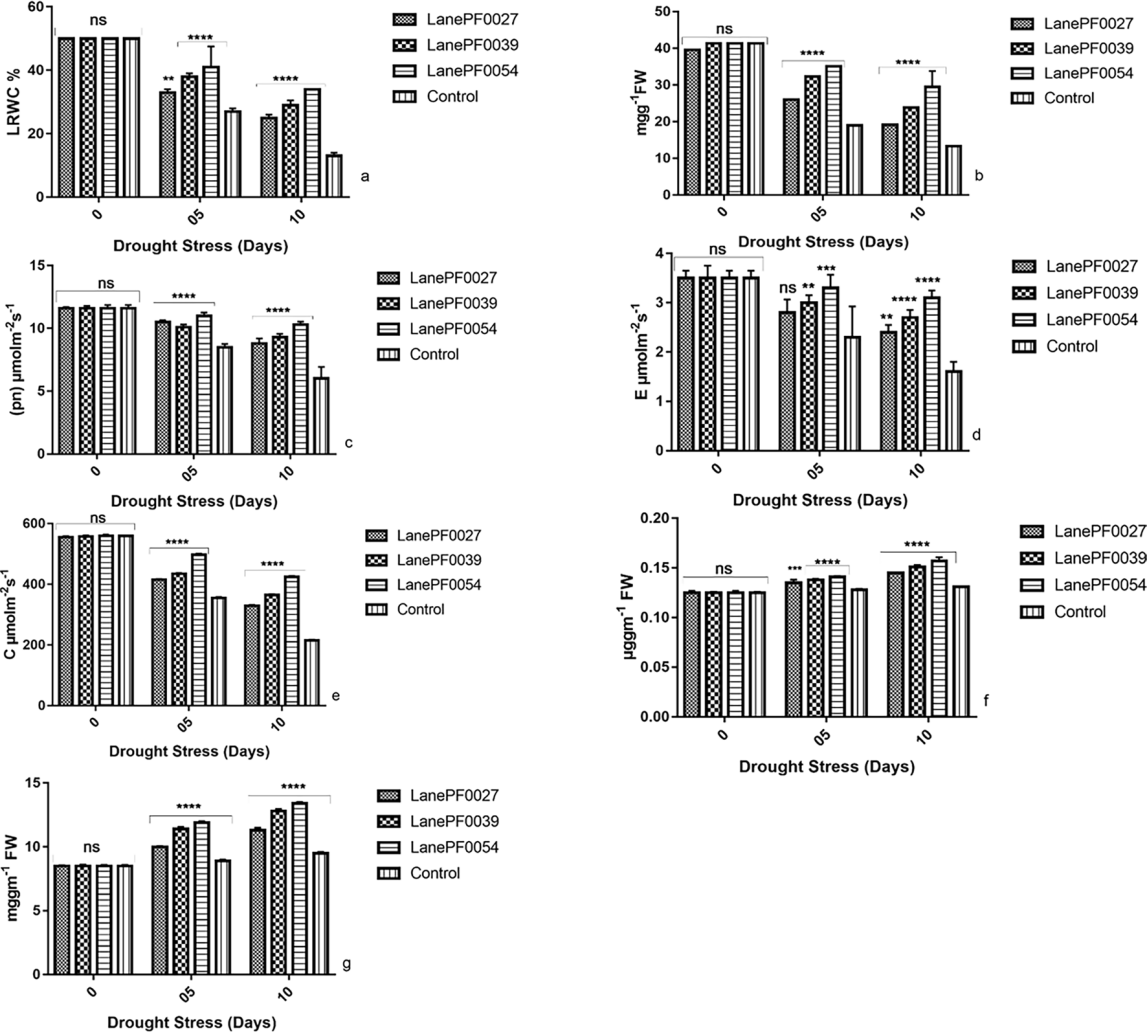
Effect of drought stress on physiological parameters of transgenic cotton plants with *GaZnF* gene. (**a**) Comparison of Leaf relative water content of transgenic and control plants, (**b**) Comparison of chlorophyll of transgenic and control plants, (**c**) Comparison of photosynthesis rate of transgenic and control plants, (**d**) Comparison of transpiration rate of transgenic and control plants, (**e**) Comparison of stomatal conductance of transgenic and control plants, (**f**) Comparison of proline content of transgenic and control plants, (**g**) Comparison of soluble sugar of transgenic and control plants. The means ± SD of three biological replicates are presented. Error bars represent the standard deviation of the mean. Above the bars ****, ***, ** represent significant and ns represent non-significant differences between transgenic and non-transgenic plants respectively at the *P* < 0.05 level according to LSD multiple range test.

Physiological parameters like leaf relative water content, chlorophyll, photosynthesis, transpiration rate and stomatal conductance, while biochemical parameters include proline and soluble sugars, are calculated to evaluate the response of *GaZnF* against drought tolerance in transgenic cotton plants as compared to non-transgenic control plants. Leaf relative water content of transgenic plants PF0054, PF0027 and PF0039 is 25%, 29% and 34% respectively as compare to non-transgenic control plant is 27% at 5 day of drought stress while at 10 day of drought stress, the transgenic plants PF0054, PF0027 and PF0039 showed 33%, 38% and 41% respectively as compared to non-transgenic control plants that is 13%. Plant Number PF0054 showed higher leaf relative water content than other two transgenic plants (Fig. 8a). Analysis of the chlorophyll content showed a substantial increase in the transgenic cotton plants as compared to the content in the non-transgenic control plants. In the transgenic cotton plants PF0054, PF0027 and PF0039 the chlorophyll content is 26.04 mgg^−1^, 32.32 mgg^−1^ and 35.08 mgg^−1^, compared to the non-transgenic control plant is 18.96 mgg^−1^. In contrast, at 10 day the maximum and minimum chlorophyll content are 29.46mgg^−1^ and 23.8mgg^−1^ in PF0054 and PF0039 respectively. While least value is calculated in PF0027 is 19.12mgg^−1^, which is also higher as compare to the non-transgenic control plant where the chlorophyll content is 13.27mgg^−1^. The transgenic cotton plants contained 10.85% more chlorophyll than the non-transgenic control plant (Fig. 8b). Photosynthesis rate is 9.7 µmolm^−2^ s^−1^, 10.1 µmolm^−2^ s^−1^ and 11 µmolm^−2^ s^−1^ as compare to non-transgenic control plant that is 8.5 µmolm^−2^ s^−1^while at 10 days photosynthesis rate is declined in transgenic plants PF0027, PF0039 and PF0054 as 8.6µmolm^−2^ s^−1^, 9.3µmolm^−2^ s^−1^ and 10.3µmolm^−2^ s^−1^ respectively as compared to non-transgenic control plant as 6.0µmolm^−2^ s^−1^. Photosynthesis in transgenic plants was better as compared to non-transgenic control plants due to the effective response of transgene (Fig. 8c). Initially transpiration rate before drought stress is3.5µmolm^−2^ s^−1^ that declined to 2.8µmolm^−2^ s^−1^, 3µmolm^−2^ s^−1^ and 3.3 µmolm^−2^ s^−1^ in PF0027, PF0039 and PF0054 at 5 day of drought stress as compared to non-transgenic control plant that is 2.3 µmolm^−2^ s^−1^. Transpiration rate further decreased to 2.4 µmolm^−2^ s^−1^, 2.7 µmolm^−2^ s^−1^ and 3.1 µmolm^−2^ s^−1^ in PF0027, PF0039 and PF0054 respectively at day10 as compared to non-transgenic control plant as 1.6 µmolm^−2^ s^−1^. A decline trend in transpiration rate was observed in transgenic and non-transgenic plants. Still, control plants are affected more than in transgenic plants (Fig. 8d). Stomatal conductance is also affected under drought stress. Still, transgenic plants showed more resistance as compared to non-transgenic control plants. Stomatal conductance of PF0027, PF0039 and PF0054 is 415 µmolm^−2^ s^−1^, 435 µmolm^−2^ s^−1^ and 498 µmolm^−2^ s^−1^ as compared to the non-transgenic control plant that is 355 µmolm^−2^ s^−1^ at 5 day of drought stress. In contrast, stomatal conductance decreased to 329 µmolm^−2^ s^−1^, 365 µmolm^−2^ s^−1^ and 425 µmolm^−2^ s^−1^ in PF0027, PF0039 and PF0054 at day 10 of drought stress as compared to non-transgenic control plant that is 215 µmolm^−2^ s^−1^ (Fig. 8e). Two-way ANOVA indicated the significant difference between the relative leaf water content, chlorophyll, photosynthesis, transpiration rate and stomatal conductance of transgenic and non-transgenic genotypes (*P* < 0.05). In counter drought stress conditions, proline values triggered for the defense of plants are more in the transgenic plant than non-transgenic control plants. Proline values at day 5 of drought stress is 7.1µggm^−1^, 9.02 µggm^−1^ and 14.3 µggm^−1^ in PF0027, PF0039 and PF0054, respectively, as compared to non-transgenic control plant that is 4.5 µggm^−1^ while at day 10 the values were 11.5 µggm^−1^, 14.9 µggm^−1^ and 18.3 µggm^−1^ in PF0027, PF0039 and PF0054 respectively as compared to non-transgenic control plant that is 6.3 µggm^−1^ (Fig. 8f). Soluble sugars also increased in response to drought stress in transgenic lines as 10 mggm^−1^, 11.4 mggm^−1^ and 11.9 mggm^−1^ in PF0027, PF0039 and PF0054 as compare to non-transgenic control plant that is 8.9 mggm^−1^ at 5 day of drought stress. In contrast, the values at 10 day of drought stress are increased to 11.3 mggm^−1^, 12.8 mggm^−1^ and 13.4 mggm^-1^ as compared to the non-transgenic control plant is 9.5 mggm^−1^ (Fig. 8g). Two-way ANOVA indicated the significant difference between the relative proline and total soluble sugar of transgenic and non-transgenic genotypes (*P* < 0.05).

## Discussion

Cotton is one of Pakistan’s GDP-contributing crops, but drought stress results in approximately 30% reduction in its yield^26^. The role of Zinc finger transcription factors is well established to regulate various cellular processes and enhance abiotic stress tolerance in transgenic plants^27,28^.The ZF-HD gene family has been identified in *Arabidopsis thaliana*, rice (*Oryza sativa*) and tomato (*Solanum lycopersicum*)^29^. It plays an important role in plants’ growth and development in response to abiotic stresses (Mao et al. 2016), Ref.^30^. ZF_HD family consist of 22 transcription factors that belong to *Gossypium arboreum* species that were divided in three main subfamilies A, B and C. Family B is further subdivided into B1, and B2 subfamilies and *GaZnF* transcription factor belongs to transcription factors A-19854 and A-27654 that are known as zinc finger homeodomain proteins and thought to be involved in the formation of homo and heterodimers and may form a zinc finger^31^. In the present study, the subcellular localization of *GaZnF* in the nucleus showed consistency with the characteristics of TFs^11^. The integration of *GaZnF* in genomic DNA of transgenic plants revealed 531 bp fragment. Stable insertion of AtNHX1 gene in *Arabidopsis thaliana* has shown and confirmed its integration and expression by Southern blot and RT-PCR, respectively^32^. Likewise, Southern blot analysis of *GbWRKY2* transcription factor was carried out to confirm its stable integration in the genome of *G. biloba*^33^. The specified expression through Western blot indicated a 95 kDa fusion band of *GaZnF* and GUS, as confirmed with anti-histidine antibody. Western blot of *bZIP* protein detected the water deficit-responsive nature of the gene^34^. Spatial expression of *GaZnF* through real-time expression analysis indicated that transgene was strongly expressed in leaf and stem under drought at vegetative and flower stages. Overall, the relative fold expression was higher in leaf tissues at the vegetative stage. Stronger relative fold expression of DREB2A and AQP7 was observed in transgenic plants compared to the control under stress^35^. The expressions of DREB2 were abundant in leaves, root tips, and root junctions, whereas the expression of AQP7 was found in root tip and root junction, but signals were barely detectable in leaves.

The transgene *GaZnF* enhanced drought tolerance in transgenic plants as determined by different growth parameters like plant height (*P* < 0.05), root-to-shoot weight ratio (*P* < 0.05), the percentage reduction in biomass (*P* < 0.05), root weight ratio (*P* < 0.05), leaf weight ratio (*P* < 0.05) and stem weight ratio (*P* < 0.05) while physiological and biochemical parameters like leaf relative water content (*P* < 0.05), chlorophyll (*P* < 0.05), photosynthesis (*P* < 0.05), transpiration rate (*P* < 0.05), stomatal conductance (*P* < 0.05), proline (*P* < 0.05) and total soluble sugar (*P* < 0.05) content respectively showed significant results under drought stress as compared to non-transgenic control plants. Altogether three transgenic plants PF0054, PF0027 and PF0039 showed drought tolerance than control non-transgenic plants. Transgenic plant PF0039 showed more drought tolerance compared to the other two transgenic plants PF0054 and PF0027. Drought tolerance is a complex agronomic trait with multi-genic components which intricately interact in plant systems^36^. The LRWC and chlorophyll content are widely used to evaluate drought stress tolerance in various plant species^37^. Accumulation of proline in transgenic plants was more as compared to non-transgenic plants. Overexpression of *GhABF2* in transgenic cotton caused higher accumulation of proline compared to wild type after drought treatment^38^. Total soluble sugar level was increased in germinating seeds of drought tolerant lentil genotypes than drought sensitive under drought stress conditions^39^. Likewise, our study showed an increasing number of soluble sugars in transgenic plants compared to non-transgenic plants under drought stress. Hence, this study concludes the transgenic cotton plants containing transcription factor gene (GaZnF) showed improvements in the morphological, physiological and biochemical characters under drought stress. The future studies for selection of homozygous lines will pave the way to breed the plants in field conditions and to select the plants with improved drought tolerance.

## Supplementary Information

Supplementary Figure S1.

Supplementary Figure S2.

Supplementary Legends.

## Data Availability

All of the data generated during research has been provided in the manuscript and its supplementary file. Data sharing not applicable to this article as no datasets generated during the current study.
