# Supplementary figures and images for: Transformation and expressional studies of GaZnF gene to improve drought tolerance in Gossypium hirsutum
Source: Sci Rep. 2023 Mar 28;13:5064. doi: 10.1038/s41598-023-32383-0 (PMC10050179; doi:10.1038/s41598-023-32383-0)

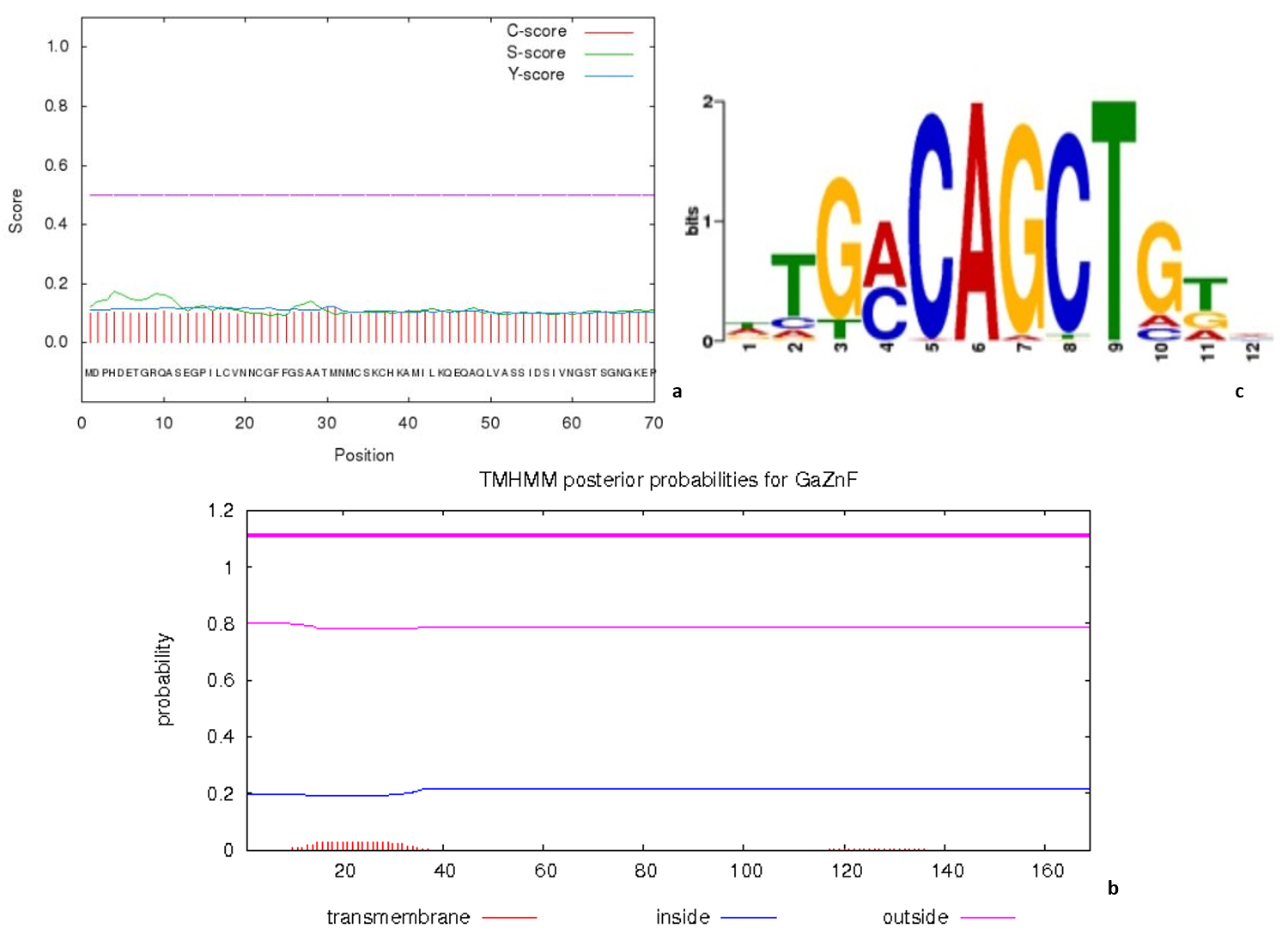

Supplement: Supplementary file 1 — Supplementary Figure S1. [file 41598_2023_32383_MOESM1_ESM.tif]

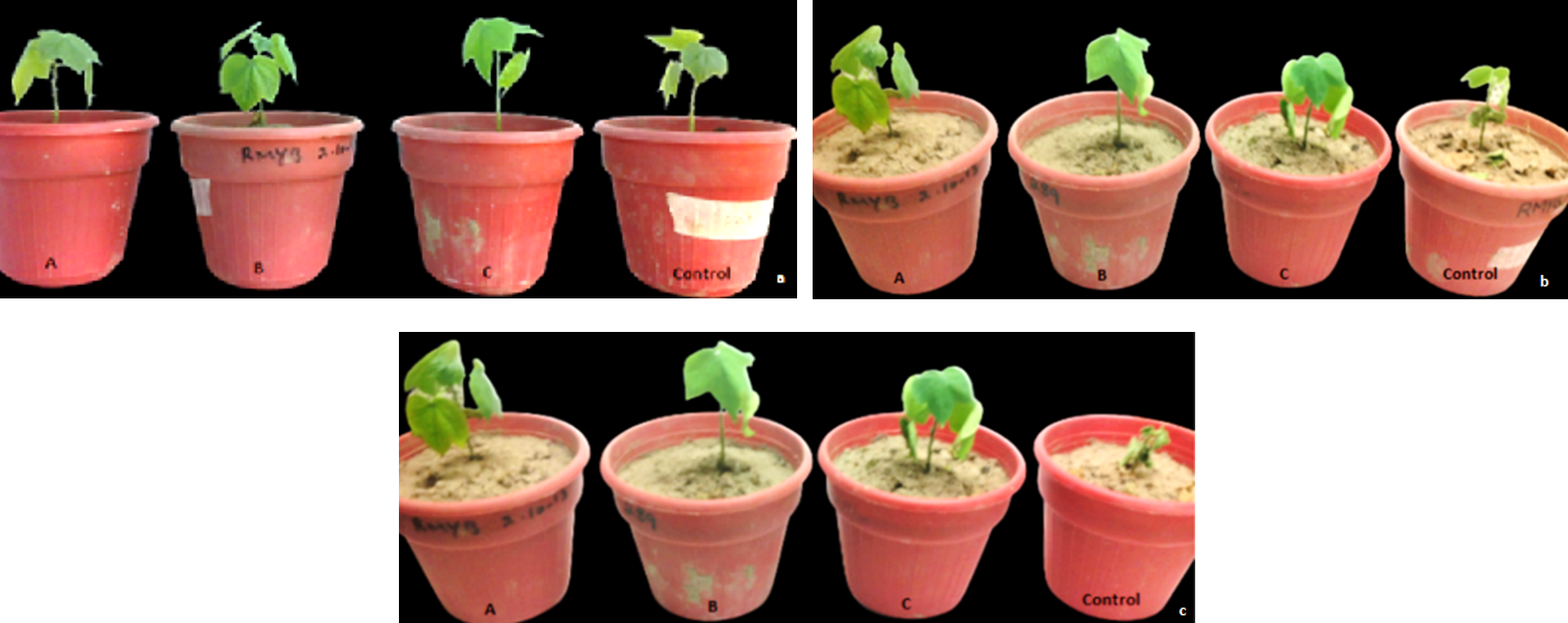

Supplement: Supplementary file 2 — Supplementary Figure S2. [file 41598_2023_32383_MOESM2_ESM.tif]
