# Supplementary material for: Transformation and expressional studies of GaZnF gene to improve drought tolerance in Gossypium hirsutum
Source: Sci Rep. 2023 Mar 28;13:5064. doi: 10.1038/s41598-023-32383-0 (PMC10050179; doi:10.1038/s41598-023-32383-0)
Supplement: Supplementary file 3 — Supplementary Legends. [file 41598_2023_32383_MOESM3_ESM.docx]

**Supplementary material**

**Transformation and Expressional studies of *GaZnF g*ene to improve drought tolerance in *Gossypium hirsutum***

**Fatima Batool^1^, Sameera Hassan^1^, Saira Azam^1^, Zunaira Sher^1^, Qurban Ali^2^*, Bushra Rashid^1^***

^1^National Centre of Excellence in Molecular Biology, University of the Punjab Lahore, 87 W Canal Bank Road Thokar Niaz Baig Lahore-53700, Pakistan

^2^Department of Plant Breeding and Genetics, Faculty of Agricultural Sciences, University of the Punjab Lahore, Pakistan

*Corresponding Author Email address: [bushra.cemb@pu.edu.pk](mailto:bushra.cemb@pu.edu.pk), [saim1692@gmail.com](mailto:saim1692@gmail.com)

**Fig 1S. Prediction of *GaZnF* transmembrane structure, signal peptide and binding motif**

**(**a) Prediction of *GaZnF* transmembrane structure (b) P *GaZnF* signal peptide prediction (c) *GaZnF* binding motif. The letters in the figure indicate bases, and the size of the letters indicates the probability of occurrence

**Fig 2S. Effect of drought stress on transgenic cotton plants with *GaZnF* gene as compare to control Plants.**

**(**a) Plants at 0 day of stress treatment (A) Lane PF0054 (B) Lane PF0039 (C) Lane PF0027 (Control) Non- Transgenic Plant **(**b) Plants at 05 day of stress treatment (A) Lane PF0054 (B) Lane PF0039 (C) Lane PF0027 (Control) Non- Transgenic Plant **(**c) Plants at 10 day of stress treatment (A) Lane PF0054 (B) Lane PF0039 (C) Lane PF0027 (Control) Non- Transgenic Plant.
